# Spatio-temporal distributions of COVID-19 vaccine doses uptake in the Netherlands: a Bayesian ecological modelling analysis

**DOI:** 10.1017/S0950268824001249

**Published:** 2024-10-07

**Authors:** Haoyi Wang, Tugce Varol, Thomas Gültzow, Hanne M. L. Zimmermann, Robert A. C. Ruiter, Kai J. Jonas

**Affiliations:** 1 Department of Work and Social Psychology, Maastricht University, Maastricht, The Netherlands; 2 Viroscience Department, Erasmus Medical Center, Rotterdam, The Netherlands; 3 Freudenthal Institute, Faculty of Science, Utrecht University, Utrecht, The Netherlands; 4 Department of Theory, Methods & Statistics, Faculty of Psychology, Open University of the Netherlands, Heerlen, The Netherlands

**Keywords:** Bayesian spatio-temporal analysis, COVID-19, prevention, small area estimation, vaccination uptake

## Abstract

In the transitioning era towards the COVID-19 endemic, there is still a sizable population that has never been vaccinated against COVID-19 in the Netherlands. This study employs Bayesian spatio-temporal modelling to assess the relative chances of COVID-19 vaccination uptake – first, second, and booster doses – both at the municipal and regional (public health services) levels. Incorporating ecological regression modelling to consider socio-demographic factors, our study unveils a diverse spatio-temporal distribution of vaccination uptake. Notably, the areas located in or around the Dutch main urban area (Randstad) and regions that are more religiously conservative exhibit a below-average likelihood of vaccination. Analysis at the municipal level within public health service regions indicates internal heterogeneity. Additionally, areas with a higher proportion of non-Western migrants consistently show lower chances of vaccination across vaccination dose scenarios. These findings highlight the need for tailored national and local vaccination strategies. Particularly, more regional efforts are essential to address vaccination disparities, especially in regions with elevated proportions of marginalized populations. This insight informs ongoing COVID-19 campaigns, emphasizing the importance of targeted interventions for optimizing health outcomes during the second booster phase, especially in regions with a relatively higher proportion of marginalized populations.

## Introduction

The coronavirus disease 2019 (COVID-19) caused by Severe Acute Respiratory Syndrome Coronavirus 2 (SARS-CoV-2) was declared a pandemic by the World Health Organization (WHO) in March 2020 [1] and is now transitioning towards an endemic status in many countries [2, 3], including the Netherlands. One of the reasons for the ‘way out of the pandemic’ was due to the fast-developed COVID-19 vaccines [2, 4].

To facilitate COVID-19 vaccination uptake in the Netherlands, great efforts have been made by the Dutch National Institute for Public Health and the Environment (RIVM), through promoting public campaigns [5, 6] and conducting behavioural studies to inform public health communication and policies [7]. Unfortunately, a sizeable part (16.5%) of the Dutch adult population still has not been vaccinated against COVID-19 according to the latest available data as of 4 September 2022 [5, 8]. Additionally, vaccine hesitancy was reported to be present in the Netherlands [9–11], which the WHO listed as one of the ten global threats to health [12].

Several determinants have been reported to be associated with individuals’ lower chance of vaccination uptake from previous studies, for example, safety concerns and side effects of vaccines, a distrust of COVID-19 vaccines and governments, having a low socio-demographic position, for example, due to belonging to an ethnic minority group, holding a low level of education, or being financially disadvantaged [13–15]. Most of these insights focus on individual risk profiles and individuals’ beliefs underlying their decision-making for vaccination uptake. Yet, to further close ‘vaccination gaps’ to prevent serious COVID-19-related health outcomes, evidence from an ecological perspective is warranted, too, such as identification of the geographical and temporal clusters of regions on a small area level and populations with a lower chance of vaccination uptake. With this information, COVID-19 vaccine-related services and interventions can be better tailored and targeted to populations with a lower chance of vaccination uptake with higher needs [4].

To investigate COVID-19 vaccination uptake on a small area level and to provide robust estimations Bayesian spatio-temporal analysis can be used. Bayesian spatio-temporal analysis is a well-established method for small-area-estimations [16–19]. Briefly, Bayesian spatio-temporal analysis can account for several sources of error or bias including spatial autocorrelation between neighbouring regions and proximity and time-dependent autocorrelation between consecutive periods in sparsely populated areas, compared to the observed frequentist prevalence calculation [17, 20, 21]. Despite Bayesian spatial and spatio-temporal analysis having been applied to monitor the distribution of COVID-19 infections in some countries, such as the United States and China [21, 22], and the distribution of COVID-19 vaccination uptake, such as in Belgium [23], it has neither been applied in the Netherlands for COVID-19 infections nor vaccination uptake monitoring.

Therefore, in this study, we aimed to apply Bayesian spatio-temporal analysis to identify clusters of lower COVID-19 vaccination uptake, among its population on the level of both the municipality and the public health services (in Dutch: Gemeentelijke of Gemeenschappelijke Gezondheidsdienst (GGD), which is a smaller regional health-specific administrative level) in the Netherlands. As a secondary objective, we investigated whether identifying clusters of lower COVID-19 vaccination uptake on a finer-defined geographical unit would reveal a more precise or different spatio-temporal pattern of COVID-19 vaccination uptake. Also, given the established evidence on how socio-demographic factors can influence individuals’ vaccination COVID-19 decisions [13–15], we aimed to explore how these socio-demographic factors may impact the overall COVID-19 vaccination uptake on an ecological level.

## Methods

### Study population

#### Study area

In the Netherlands, the planning, monitoring, and evaluation of public health measures, including COVID-19 vaccination, are mostly carried out by the GGDs. The Netherlands has 25 GGD regions in total. Within the GGD regions, the smallest administrative units are on the municipality level, which entails 345 municipalities in total. Estimates on both GGD and municipality levels provide valuable information for Dutch policymakers [20].

#### Data sources

We retrieved surveillance data on COVID-19 vaccination uptake, by vaccination scenarios, from RIVM with the openly accessible COVID-19 vaccination uptake data [5]. For the beginning phase of the vaccination promotion, due to the requirement of privacy protection by RIVM, data on the vaccination prevalence in regions with less than 5% coverage were masked. We assumed a 0% coverage for these regions.

For the socio-demographic spatial proxies, we retrieved freely accessible data per municipality/GGD from Statistics Netherlands (CBS), which provides reliable statistical information across the Netherlands to produce insight into social issues [24], and linked them with the COVID-19 vaccination uptake surveillance data on the areal level. The following datasets were used in this study: (a) ‘Bevolking 15 tot 75 jaar; opleidingsniveau, wijken en buurten (Population 15 to 75 years; education level, districts and neighbourhoods)’ for data on the proportion of low education, 2019 [25]; (b) ‘Bevolking; migratieachtergrond, generatie, leeftijd, regio, 1 januari 2021 (Population; migration background, generation, age, region, 1 January 2021)’ for data on the proportion of non-western migration background population [26]; and (c) ‘Kerncijfers wijken en buurten 2021 (Key figures for districts and neighbourhoods 2021)’ for data on the proportion of the financially extremely disadvantaged population [27].

#### Study population and vaccination scenarios

We included data from all populations who were 18 years and older in this study. In the Netherlands, five different vaccines have been used: Moderna (Spikevax), BioNTech/Pfizer (Comirnaty), AstraZeneca (Vaxzevria), Janssen, and Novavax [5]. Given that the majority of the administrated COVID-19 vaccines were the 2-doses based mRNA COVID-19 vaccines, such as Pfizer/BioNTech and Moderna [28], we conducted analyses on three different COVID-19 vaccination scenarios (hereinafter vaccination scenarios), namely (1) covered primary partly (only one dose of the selected vaccine has been administered), (2) covered primary completed (two doses of the selected vaccine have been administered), and (3) covered first booster (covered primary completed and one booster dose), following the definitions and terminologies from RIVM [5]. Only a minority of the Dutch population has been vaccinated against COVID-19 using the 1-dose-based viral vector COVID-19 vaccine, Janssen [28]. For this population, following RIVM’s recommendation, we considered administrating one dose as primarily completed [29].

#### Study periods

The first COVID-19 vaccines were administered on 6 January 2021, in the first-dose vaccination promotion in the Netherlands [5]. After the first-dose vaccination promotion, the second-dose promotion and the booster-dose proportion were started in February 2021 and November 2021, respectively. To achieve maximum vaccination uptake [6], in the Dutch vaccination program, populations with clinical health vulnerabilities were vaccinated first, followed by stratification based on age groups from the elderly to the young. Given the different vaccination-promoting periods, to avoid misleading spatio-temporal interaction estimation, we only retrieved the periods when the COVID-19 vaccination was available to our total selected study population. As a result, we only included data on COVID-19 vaccination covered primary partly from February 2021 to August 2022 (19 months in total); on COVID-19 vaccination covered primary completed from March 2021 to August 2022 (18 months in total), and on COVID-19 vaccination covered first booster from November 2021 to August 2022 (10 months in total).

### Bayesian spatio-temporal analysis

To estimate the relative chance of COVID-19 vaccination uptake on the small area levels (municipality and GGD levels), we used the Integrated Nested Laplace Approximation (INLA), which is a computationally less intensive but efficient and equivalent alternative to Markov chain Monte Carlo for the Bayesian computation [19]. We appointed a Penalized Complexity (PC) prior to the precision of the exchangeable random effects, by employing the re-parameterised Besag-York-Mollie (BYM2) model [30]. This model specifies the spatially structured residual using an intrinsic conditional autoregressive distribution [30, 31], and uncertainties due to the instability of estimates in sparsely populated areas [16, 19], assuming the regional characteristics are more similar by proximity than by distance, as informed by the spatial connectivity outlined in Supplementary Figure S1.

In this study, we first applied this method to describe the spatio-temporal relative chance of COVID-19 vaccination uptake by the vaccination scenarios and two geographical levels with a space-time interaction to explore the spatio-tempotal trends. To further understand the spatial trends of the relative chance of COVID-19 vaccination dose uptake in each selected period, we also investigated the spatial relative chance of COVID-19 vaccination uptake by the vaccination scenarios and two geographical levels over the selected periods without the space-time interaction. These estimates indicate the spatial distribution of the COVID-19 vaccination uptake at one certain timepoint (for results in Supplementary Materials S8–S13).

### Spatio-temporal ecological modelling

In this study, we also explored whether COVID-19 vaccination uptake in the Netherlands was influenced by the selected regional socio-demographic characteristics as the spatial proxies on an ecological level. We, therefore, applied a spatio-temporal ecological regression modelling technique [19] which takes these spatial proxies into account to pick up additional associations and noises [17]. For the proportion of non-Western migrants and the proportion of financially extremely disadvantaged individuals. Data were retrieved from 2021, assuming these two spatial proxies were stable for the selected periods in this study. For the variable of non-Western migrants, we considered individuals originating from a country in Africa, South America or Asia (excl. Indonesia and Japan) or from Turkey as non-Western migrants following the CBS’s definition. For the variable of the proportion of financially extremely disadvantaged individuals, we used data on people who were taking Bijstandsuitkering, a subsidy that is only available for those living with less than minimum wage or who cannot sustain themselves. For data on the proportion of individuals with a low level of education, we considered people who do not have a high school or equivalent diploma as having a low level of education, following the CBS’s definition. We then extrapolated data from 2019, given that the most recent data was only available from 2019 from CBS. We assumed that the proportion of individuals with a low level of education from 2019 is comparable to 2021–2022 and stable over time.

We first conducted univariable models which only included one of the selected spatial proxies and the space-time interaction. The models’ goodness of fit was assessed using the deviance information criterion (DIC). With consistent random effect parameter structures across our models and a limited set of predictors (three in total), we aimed to comprehensively explore potential ecological associations. Therefore, we proceeded by conducting multivariable models incorporating all determinants found to be significant in the univariable models, as indicated by Bayesian credible intervals (CrIs) [32]. CrIs can be regarded as a Bayesian analogue to confidence intervals to present the 95% probability of the posterior means. All results on the spatio-temporal distribution of COVID-19 vaccination uptake presented in this study were based on the spatio-temporal final regression models.

For models’ assumptions and parameters’ appointments, see Supplementary Material S1. All analyses were conducted in R (version 4.3.2).

## Results

### Spatio-temporal trends of COVID-19 vaccination uptake in the Netherlands

**Figure 1.**
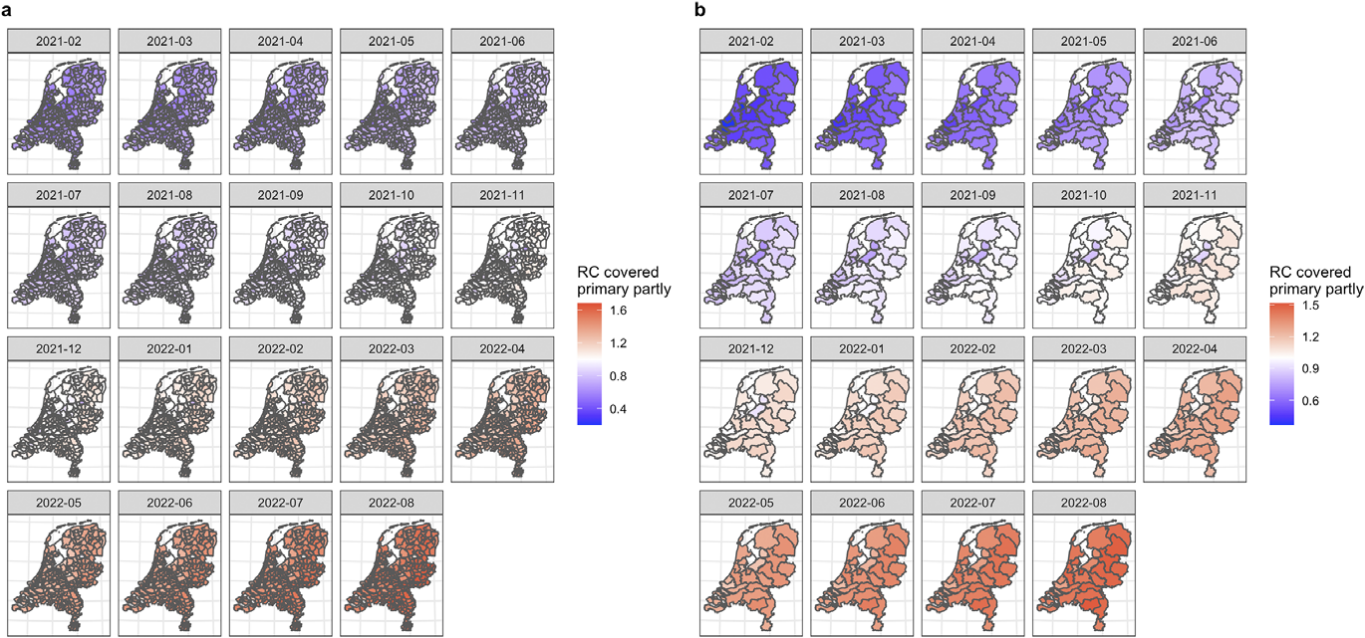
Choropleth map of the relative chance of COVID-19 vaccination uptake covered primary partly (only one dose administrated) on (a) municipality level and (b) Public health services (GGD) level by Bayesian spatio-temporal ecological modelling (final model), February 2021 to August 2022. *Note:* RC, relative chance, * indicates results estimated by Bayesian spatio-temporal ecological final model. For better visibility, larger figures can be found in Supplementary Materials S1–S2. RC higher than 1 indicates a higher-than-average (average risk in the Netherlands) chance of COVID-19 vaccination in that region (red); RC lower than 1 indicates a lower-than-average chance of COVID-19 vaccination in that region (blue).

**Figure 2.**
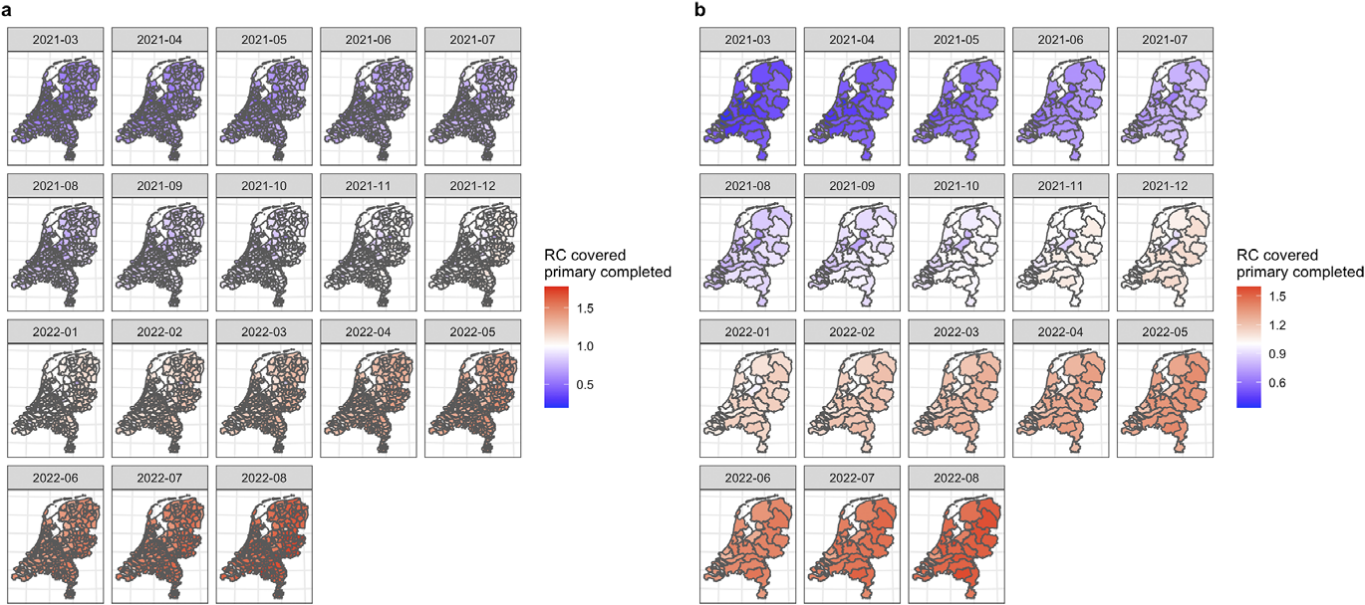
Choropleth map of the relative chance of COVID-19 vaccination uptake covered primary completed (two doses administrated) on (a) municipality level and (b) Public health services (GGD) level by Bayesian spatio-temporal ecological modelling (final model), March 2021 to August 2022. *Note:* RC, relative chance, * indicates results estimated by the Bayesian spatio-temporal ecological final model. For better visibility, larger figures can be found in Supplementary Materials S3–S4. RC higher than 1 indicates a higher-than-average (average risk in the Netherlands) chance of COVID-19 vaccination in that region (red); RC lower than 1 indicates a lower-than-average chance of COVID-19 vaccination in that region (blue).

**Figure 3.**
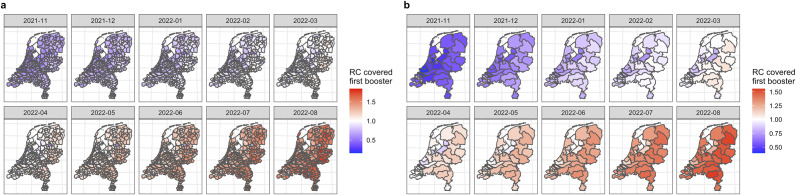
Choropleth map of the relative chance of COVID-19 vaccination uptake covered first booster (one booster dose administrated) on (a) municipality level and (b) Public health services (GGD) level by Bayesian spatio-temporal ecological modelling (final model), November 2021 to August 2022. *Note:* RC, relative chance, * indicates results estimated by the Bayesian spatio-temporal ecological final model. For better visibility, larger figures can be found in Supplementary Materials S5–S6. RC higher than 1 indicates a higher-than-average (average risk in the Netherlands) chance of COVID-19 vaccination in that region (red); RC lower than 1 indicates a lower-than-average chance of COVID-19 vaccination in that region (blue).

Figures 1– 3 present the spatio-temporal distribution of the relative chance of COVID-19 vaccination uptake covered partially, completed, and boosted, respectively, on the (a) municipality level and (b) GGD level, compared to the average chance of vaccination uptake at the national level, from February 2021 to August 2022. The corresponding spatial distribution of the relative chance of these scenarios at each selected period can be found in Supplementary Materials S8–S13.

Spatially, no major differences in the spatial trends of these scenarios were found between the spatio-temporal models and the spatial models. Temporally, the spatio-temporal models estimated a steady increase in the relative chance of COVID-19 vaccination uptake for all regions and vaccination scenarios over the selected periods.

#### COVID-19 vaccination covered primary partly uptake, February 2021 to August 2022

Of the included periods, we identified a similar spatio-temporal trend of the COVID-19 vaccination uptake covered primary partly on both municipality and GGD levels. In general, spatially, of each selected temporal period, the highest relative chance of the COVID-19 vaccination uptake covered primary partly was found in the East of the Netherlands, while the lowest relative chances were found in the West, especially among municipalities close to the in the areas located in or around the main urban areas (in Dutch ‘Randstad’, which entails the agglomeration of cities in the west of the Netherlands, in particular, Amsterdam, Utrecht, Leiden, The Hague, and Rotterdam [33]).

We also identified internal heterogeneity of the relative chances among municipalities within the GGD level over time. Taking the GGD Amsterdam region as an example, the overall relative chance of the COVID-19 vaccination uptake covered primary partly in February 2021 was estimated to be 0.368 (95% CrI 0.367;0.269). Within the GGD Amsterdam region, the relative chance among municipalities ranged from 0.374 (0.373;0.375) in Amsterdam to 0.614 (0.607;0.621) in Ouder-Amstel; and the overall relative chance in August 2022 was estimated to be 1.316 (1.314;1.318). The relative chance among municipalities ranged from 1.317 (1.314;1.319) in Amsterdam to 1.560 (1.551;1.569) in Aalsmeer.

#### COVID-19 vaccination covered primary completed, March 2021 to August 2022

We identified similar spatio-temporal trends of the relative chance on both geographical levels for this vaccination scenario, too. What is noteworthy to mention is that lower relative chances of COVID-19 vaccination uptake among individuals who have completed both doses were identified in regions spanning from the Southwest to the Northeast, which is considered the agglomeration of more religiously conservative regions in the Netherlands.

#### COVID-19 vaccination covered first booster, November 2021 to August 2022

Again, similar spatio-temporal trends with internal spatial heterogeneity within the GGD regions were identified compared to the COVID-19 vaccination uptake covered primary partly and completed. Regions located in or around the Randstad region and regions that are more religiously conservative showed a lower relative chance of the COVID-19 vaccination uptake covered first booster compared to other regions in the Netherlands over the selected periods.

In addition, in this vaccination scenario, we found that, on the GGD regional level, the GGD-Zuid-Limburg region, for example, showed a lower relative chance of the COVID-19 vaccination uptake covered first booster over the selected periods as well. Taking the median selected period of March 2022 as an example, the overall relative chance of the COVID-19 vaccination uptake covered first booster was estimated to be 0.953 (0.951;0.955), which can be considered as a significantly lower relative chance compared to the average national chance over the selected periods. While zooming in on the municipality level, the relative chances ranged from 0.726 (0.718;0.734) in Vaals to 1.148 (1.141;1.154) in Eijsden-Margraten which presented a significantly higher chance compared to the average chance of vaccination uptake on the national level.

For detailed relative chance per region and per selected period of each vaccination scenario on both geographical levels, see Supplementary Excel File 1.

### Bayesian ecological modelling on spatial socio-demographic proxies on COVID-19 vaccination uptake by COVID-19 vaccination scenarios

#### Univariable models

All detailed regional spatial socio-demographic proxies’ characteristics can be found in Supplementary Excel File 2. Univariably, for the three COVID-19 vaccination uptake scenarios, a higher proportion of non-Western migrants and a higher proportion of financially extremely disadvantaged individuals were associated with a lower chance of COVID-19 vaccination uptake on both geographical levels significantly.

#### Multivariable models

After adjusting for all the significant spatial socio-demographic proxies identified in the univariable model, in the multivariable model, only the proportion of non-Western migrants was found to be significantly negatively associated with the relative chance of COVID-19 vaccination uptake of all three vaccination scenarios on both geographical levels. Taking the COVID-19 vaccination uptake covered first booster on the municipality level as an example, for each 1% increase of the proportion of non-Western migrants in one municipality, the relative chance of the booster COVID-19 vaccination in that municipality decreased by 0.9% over the selected periods.

All univariable and multivariable models affirmed the observed significantly increasing temporal trends. Taking the COVID-19 vaccination uptake covered first booster on the municipality level as an example, the relative chance of the first booster COVID-19 vaccination uptake increased by 9.7% (=exp(0.093)) for each period on average over the selected periods. For all detailed results obtained from other uni-/multivariable models for all three vaccination scenarios on both geographical levels, see Table 1.

**Table 1.** Model summary of Bayesian spatio-temporal ecological analysis of COVID-19 vaccination uptake in the Netherlands by vaccination scenarios

Vaccination scenarios	Models	Covariates	Municipality level analyses					Public health services level analyses				
			Coefficient	95% CrI	Temporal period	95% CrI	DIC (Difference from null model)	Coefficient	95% CrI	Temporal period	95% CrI	DIC (Difference from null model)
Covered primary partly (one dose administrated)	Null model	–	–	–	0.051	(0.051;0.052)	11932673.64 (NA)	–	–	0.064	(0.061;0.067)	3809637.23 (NA)
	Univariable models	Low education (%)	−0.175	(−0.386;0.035)	0.049	(0.049;0.050)	11930910.92 (−1762.72)	0.063	(−1.362;1.487)	0.054	(0.052;0.056)	3809636.46 (−0.77)
		Non–Western migrant (%)	−**0.864**	(−1.042;−0.687)	0.049	(0.049;0.050)	11931348.24 (−1325.4)	−**0.941**	(1.305;−0.577)	0.054	(0.052;0.056)	3809642.82 (5.59)
		Financially extremely disadvantaged (%)	−**4.521**	(−5.839;−3.202)	0.049	(0.049;0.050)	11931092.45 (−1581.19)	−**8.548**	(−14.173−2.895)	0.054	(0.052;0.056)	3809639.14 (1.91)
	Multivariable model	Low education (%)	NA	NA	0.049	(0.049;0.050)	11931265.87 (−1407.77)	NA	NA	0.054	(0.052;0.056)	3809641.70 (4.47)
		Non–Western migrant (%)	−**0.774**	(−1.010;−0.538)				−**0.906**	(−1.431;−0.378)			
		Financially extremely disadvantaged (%)	−0.958	(−2.613;0.697)				−0.609	(−0.7171;5.930)			
Covered primary completed (two doses administrated)	Null model	–	–	–	0.061	(0.061;0.061)	13626026.19 (NA)	–	–	0.077–	(0.074;0.080)	5182644.31
	Univariable models	Low education (%)	−0.169	(−0.386;0.049)	0.059	(0.058;0.059)	13625208.31 (−817.88)	0.222	(−1.289;1.729)	0.065	(0.063;0.067)	5182643.33 (−0.98)
		Non–Western migrant (%)	−**0.891**	(−1.076;0.706)	0.059	(0.058;0.059)	13624705.25 (−1320.94)	−**1.067**	(−1.419;−0.714)	0.065	(0.063;0.067)	5182652.84 (8.53)
		Financially extremely disadvantaged (%)	−**4.717**	(−6.083;−3.350)	0.059	(0.058;0.059)	13624434.02 (−1592.17)	−**10.883**	(−17.419;−4.205)	0.065	(0.063;0.067)	5182647.15 (2.84)
	Multivariable model	Low education (%)	NA	NA	0.059	(0.058;0.059)	13624624.22 (−1401.97)	NA	NA	0.065	(0.063;0.067)	5182651.68 (7.37)
		Non–Western migrant (%)	−**0.786**	(−1.031;−0.540)				−**0.954**	(−1.457;−0.448)			
		Financially extremely disadvantaged (%)	−1.117	(−2.837;0.604)				−1.987	(−9.292;4.293)			
Covered first booster (one booster dose administrated)	Null model	–	–	–	0.094	(0.093;0.095)	8542369.00 (NA)	–	–	0.120	(0.116;0.124)	4619293.19
	Univariable models	Low education (%)	−**0.396**	(−0.666;−0.126)	0.093	(0.092;0.093)	8540986.40 (−1382.6)	0.085	(−1.444;1.615)	0.100	(0.098;0.103)	4619291.70 (−1.49)
		Non–Western migrant (%)	−**0.985**	(−1.222;−0.748)	0.093	(0.092;0.093)	8541295.45 (−1073.55)	−**0.937**	(−1.369;−0.508)	0.100	(0.098;0.103)	4619296.28 (3.09)
		Financially extremely disadvantaged (%)	−**5.307**	(−7.036;−3.577)	0.093	(0.092;0.093)	8541082.35 (−1286.65)	−**8.393**	(−14.756;−2.008)	0.100	(0.098;0.103)	4619286.72 (−6.47)
	Multivariable model	Low education (%)	−0.250	(−5.27;0.028)	0.093	(0.092;0.093)	8541190.15 (−1178.85)	NA	NA	0.100	(0.098;0.103)	4619293.40 (0.21)
		Non–Western migrant (%)	−**0.911**	(−1.232;−0.590)				−**0.918**	(−1.534;−0.300)			
		Financially extremely disadvantaged (%)	−0.483	(−2.912;1.946)				−0.349	(−8.081;7.331)			

*Note:* CrI, credible interval; DIC, deviance information criterion; NA, not applicable. Bold values indicate significant coefficients.

## Discussion

This study explored the spatio-temporal distribution of the relative chance of COVID-19 vaccination uptake in the Netherlands with different COVID-19 vaccination scenarios ranging from covering primary partly (first dose) when the vaccines were just becoming available, over covering primary completed (second dose), to covering the first booster. We made use of the publicly available surveillance data of COVID-19 vaccination uptake which was routinely collected by RIVM. We applied Bayesian spatio-temporal modelling analysis for robust estimations accounting for spatial random effects and random noise across the spatio-temporal structure of the Netherlands on both the GGD and municipality levels. The known socio-demographic determinants of COVID-19 vaccination uptake were considered as socio-demographic spatial proxies to further fine-tune the estimates of the relative chance of vaccination uptake. As a result, COVID-19 campaigns and interventions can be better targeted to further close the vaccination gaps in the Netherlands.

### Spatio-temporal distribution of COVID-19 vaccination uptake

Overall, we observed a higher relative chance of COVID-19 vaccination uptake from the East and South of the Netherlands over the time periods included in this study for all three vaccination scenarios. The lowest relative chances of COVID-19 vaccination were identified in the areas located in or around the Randstad region and the regions that are more religiously conservative by both the spatio-temporal models and spatial models (Supplementary Materials S8–S13).

It was within our expectation that people living in areas located around more religious conservative regions would have a lower chance to take the COVID-19 vaccines, given the strong evidence of a lower chance of overall vaccination uptake in this region due to religious beliefs [34–37]. Our findings thus confirmed a lower chance of COVID-19 vaccination uptake in this region too. We suggest more efforts and behavioural interventions should be allocated to this region, such as attention to the engagement of trusted religious leaders and spokespeople [4, 38]. Also, a needs assessment to gather information regarding the problem should be conducted, which would probably lead to more effective interventions through improved targeted public health communication [39].

However, our study revealed that residents in areas located in or around the Randstad region exhibited a lower relative chance of COVID-19 vaccination uptake compared to the national average. One reason may be the higher density of non-Western migrants in the area, as indicated by prior individual-level studies in the Netherlands [10, 40] and elsewhere [15, 40, 41]. Our results from the ecological modelling analysis corroborated this, showing that a higher proportion of non-Western migrants was associated with diminished vaccination uptake over time at the population level. Future vaccination campaigns should address this group specifically. However, as ‘non-Western migrants’ encompass diverse ethnicities and cultures, targeted programs improving vaccination uptake should be nuanced and tailored based on thorough needs assessments [10]. Another possible reason contributing to the observed lower vaccination rate may be the potential underestimation of our estimates. The national COVID-19 vaccination surveillance in the Netherlands only accounts for vaccines administered within the country, not those obtained abroad. Given the significant migrant population in the Randstad region in general [42], our estimations may be conservative, overlooking vaccinations acquired outside the Netherlands.

When comparing the estimations between the GGD level and the municipality level, we demonstrated that refining the geographical scale can lead to enhanced insights. Spatial patterns of the lowest relative chance of COVID-19 vaccination uptake vary based on spatial units, showcasing distinct relative chances among municipalities within a GGD region. Take GGD-Zuid-Limburg as an example, a higher-than-average chance of COVID-19 vaccination uptake was observed in the Northwest municipalities over time, while municipalities located in the Southeast of GGD-Zuid-Limburg had a lower-than-average chance compared to the national level. This finding thus indicates the potential ecological fallacy in the ecological study. By aggregating data from a finer-defined geographic scale to a larger geographic scale, the internal heterogeneity within one larger geographic area, in our case municipalities nested within a GGD region, may not be captured and may lead to missing opportunities for public health actions and interventions. Given the GGDs’ regional health responsibility, monitoring at a finer area level is recommended to identify and address potential public health concerns promptly.

### Spatial socio-demographic proxies of COVID-19 vaccination uptake

In line with previous evidence which investigated how socio-demographic characteristics can influence one’s COVID-19 vaccination uptake on an individual level [13], our study confirmed that accounting for socio-demographic characteristics on the areal level can be helpful and could be applied as a spatial proxy for the COVID-19 vaccination uptake, too.

Throughout the COVID-19 scenarios (cover primary partially, cover primary completed and cover first booster), and on both GGD and municipality levels, we found that the proportion of non-Western migrants was associated with a lower relative chance of COVID-19 vaccination uptake in the Netherlands on the ecological level. Our results are thus in line with the previous synthesized findings for the impacts of being a non-Western migrant on COVID-19 vaccination lower uptake based on the individual level investigations [43].

Noteworthy is that even though the proportion of financially extremely disadvantaged individuals and the proportion of people with a low level of education were associated negatively with a higher chance of vaccination uptake in the univariable models significantly, this finding disappeared in the multivariable models. One of the reasons could also be that non-Western migrants more often face financial difficulties and generally also often have a lower level of education in the Netherlands [44], therefore the impact of both might have disappeared due to adjusting for the proportion of non-Western migrants on the population level. However, individual socio-demographic characteristics and their interactions with the environment are often assumed to play a bigger role in explaining health behaviours [20] and should therefore be considered in modelling above areal proxies whenever possible. Therefore, given the significance of being ‘financially extremely disadvantaged’ in the univariable models, its ecological negative impact on vaccination uptake in the Netherlands should be acknowledged, too.

Also, the fact that our estimates for the impact of the spatial proxies differ/disappeared from the municipality level to the GGD level, indicated ecological fallacy, again, given that the spatial covariates across the municipalities provided more information compared to the GGD level.

Yet, our ecological findings still indicate that sub-populational needs from non-Western migrants living in the Netherlands for additional COVID-19 vaccination services and efforts are not being fully met. Public health authorities and vaccination programmes could thus use our findings when designing future vaccination strategies for COVID-19 and other infectious diseases, such as mpox [45], by prioritizing resources and services allocation and improving public health communication to regions and sub-populations that currently have a lower chance of vaccination uptake. This study thus helps in informing the (COVID-19-)vaccine-related intervention planners about whom and where to target in particular.

### Strength and limitations

This study’s major strength is the application of Bayesian spatio-temporal modelling to three vaccination scenarios in the Netherlands, providing robust estimations of low-risk clusters of COVID-19 vaccination uptake across diverse geographical units. Incorporating a weakly informative PC prior appointment enhances precision at the small area level by accounting for space-time autocorrelation. Additionally, the study pioneers an ecological assessment of socio-demographic characteristics at a small area level in the Netherlands, offering valuable insights for current and future vaccination strategies amid infectious disease outbreaks.

We acknowledge the following limitations of our study. Firstly, our use of secondary surveillance data on COVID-19 vaccination in the Netherlands, mandated to mask proportions below 5%, may introduce partial bias in our spatial modelling analysis. However, Bayesian spatio-temporal analysis mitigates this limitation by accounting for space-time autocorrelation, minimizing potential bias in our estimations. Secondly, our data only includes adults, excluding those under 18 due to delayed approval for COVID-19 vaccines in minors. Future studies should address this gap, considering the influence of guardians’ beliefs on vaccination in minors. Thirdly, using an aggregated ‘non-Western migrant’ variable may overlook ethnic clusters, necessitating future studies to explore specific ethnic groups when accurate data are available. Fourthly, our model showed that certain regions had a lower relative chance of vaccination uptake which aligned with the more religious conservative regions in the Netherlands. However, we could not include regional religion data in our ecological models because this data is not accessible on the GGD and municipal level. Consequently, this suggestion may not be as robust as other suggestions with ecological modelling evidence, such as the proportion of non-Western migrants. We, therefore, recommend that future studies incorporate such information into their models once regional religion data becomes accessible. This inclusion will strengthen the robustness of suggestions provided by the model. Lastly, our data lacks information on the uptake of specific COVID-19 vaccines, hindering spatial and temporal identification of vaccine clusters. Future investigations should include this information for more robust evidence in advanced mathematical modelling of the COVID-19 pandemic.

## Conclusions

In conclusion, we showed that the relative chance of COVID-19 vaccination uptake was heterogeneous for all three rounds of vaccination scenarios over time in the Netherlands on both GGD and municipality levels. Estimations on the municipality level show vaccination variability and more concise clustering patterns compared to the GGD level and thus provide more insights into the strategies for vaccination services. We identified regions, such as those that are more religiously conservative, and those with a higher proportion of non-Western migrants or more financially disadvantaged, that had a lower chance of COVID-19 vaccination uptake. In the transitioning era towards the COVID-19 endemic, our results should inform public health professionals that more efforts are needed to reach individuals living in these regions in the Netherlands for the ongoing second COVID-19 booster campaign to prevent more serious COVID-19-related health outcomes. Our findings can also provide valuable information for other infectious diseases to further close the vaccination gaps in general.

## Supporting information

Wang et al. supplementary material 1 Wang et al. supplementary material

Wang et al. supplementary material 2 Wang et al. supplementary material

Wang et al. supplementary material 3 Wang et al. supplementary material

## Data Availability

The data that support the findings of this study are available from the corresponding author upon reasonable request.

## Supplementary material

The supplementary material for this article can be found at http://doi.org/10.1017/S0950268824001249.
