# Supplementary material for: Spatio-temporal distributions of COVID-19 vaccine doses uptake in the Netherlands: a Bayesian ecological modelling analysis
Source: Epidemiol Infect. 2024 Oct 7;152:e119. doi: 10.1017/S0950268824001249 (PMC11474892; doi:10.1017/S0950268824001249)

# Spatio-temporal distributions of COVID-19 vaccine doses uptake in the Netherlands: A Bayesian ecological modelling analysis

# Supplementary materials

Haoyi Wang^1,2§^, Tugce Varol^1,3^, Thomas Gültzow^1,4^, Hanne M. L. Zimmermann^1,5^, Robert A.C. Ruiter^1^, Kai J. Jonas^1^

^1^Department of Work and Social Psychology, Maastricht University, Maastricht, The Netherlands

^2^Viroscience Department, Erasmus Medical Centre, Rotterdam, The Netherlands

^3^Freudenthal Institute, Faculty of Science, Utrecht University, Utrecht, the Netherlands

^4^Department of Theory, Methods & Statistics, Faculty of Psychology, Open University of the Netherlands, Heerlen, the Netherlands

^5^Department of Infectious Diseases, Public Health Service of Amsterdam, Amsterdam, The Netherlands

^§^Correspondence to: Haoyi Wang

Department of Work and Social Psychology, Maastricht University, Maastricht, 6200ER, the Netherlands

Phone: +31 43 38 84531

Email: [haoyi.wang@maastrichtuniversity.nl](mailto:haoyi.wang@maastrichtuniversity.nl)

## S1 Model assumptions and parameter appointments for the spatio-temporal analysis of the relative chance of COVID-19 vaccination uptake in the Netherlands

In this study, for the counted outcome of vaccination uptake, we assumed that the observed vaccinated cases follow a Poisson distribution with mean $E_{i}\theta_{i}$, following the suggestions from Diggle (1983) on spatial analysis for disease mapping [1], where $E_{i}$ is the expected number and $\theta_{i}$ is the relative chance in each region $i$. We thus can define the posterior relative chance on the logarithmic scale:

$$Y_{i}\sim Po(E_{i}\theta_{i}),\text{ }i=1,\ldots,N,$$

$$E_{i}=r^{(s)}n^{(i)},$$

where $r^{(s)}$ is the prevalence of COVID-19 vaccination uptake in the Netherlands, and $n^{(i)}$ is the size of the population of region $i$. Additionally, we added a temporal dimension into the model with a spatio-temporal interaction, so the model was formed as:

where stands for the space-time interaction term:

$$u_{i}+v_{i}\boldsymbol{=b}=\frac{1}{\sqrt{\tau_{b}}}(\sqrt{1-\phi}\boldsymbol{v}_{\mathbf{*}}+\sqrt{\phi}\boldsymbol{u}_{\mathbf{*}})$$

Where $\alpha$ represents the overall chance of the vaccination uptake in the Netherlands, $\mathcal{ui}$ is a structured random effect on area $\mathcal{i}$ which is used to model spatial dependence between the relative chance, and $\mathcal{vi}$ represents other unstructured noise which follows a distribution of $v_{i}\sim N\left( 0,\sigma_{v}^{2} \right)$. $\tau_{b}>0$ is a marginal precision parameter contribution from spatial term $\boldsymbol{u}_{\mathbf{*}}$ and random effect $\boldsymbol{v}_{\mathbf{*}}$, and the fraction of this variance explained by the from spatial term $\boldsymbol{u}_{\mathbf{*}}$ and random effect $\boldsymbol{v}_{\mathbf{*}}$ are the mixing parameter $0\leq\phi\leq1$.

In spatio-temporal ecological analysis, we first applied the univariable models which only include one of the selected determinants:

where $\beta_{1}$ is the coefficient for the one of the determinants $d_{i}$ selected in this study. In the final model, we included all the significant determinants indicated by the univariate models:

$$\boldsymbol{d}_{i}=(1,d_{i1},\ldots,d_{ip})$$

$$\boldsymbol{\beta}=(\beta_{0},\beta_{1},\ldots,\beta_{p})^{'}$$

To define the PC prior, we used the probability statement $P((1/\sqrt{\tau_{b}})>U)=\alpha$. Based on the rule of thumb by Simpson et al. [2], we set $U=0.5/0.31$ and $a=0.01$. We then defined the prior for the mixing parameter $\phi$ as $P(\phi<0.5)=2/3$, which assumed that the unstructured random effect accounts for more of the variability than the spatially structured effects [3].

All analyses were conducted in R (version 4.2.1). We used SpatialEpi R package (version 1.2.7) to estimate the expected COVID-19 vaccination uptake per region [4]. For all Bayesian modelling analyses with INLA, we used the R-INLA package (version 21.05.02) [5].

## Ethical statement

The study only used publicly accessible secondary data from the national public surveillance system. Ethical approval and informed consent were therefore not required, given secondary data were depersonalised. Data was presented in an aggregated manner excluding the possibility of identifying specific individuals.

## Reference

1. Diggle P. Statistical analysis of spatial point patterns. London ;: Academic Press; 1983.

2. Simpson D, Rue H, Riebler A, Martins TG, Sorbye SH. Penalising Model Component Complexity: A Principled, Practical Approach to Constructing Priors. Statist Sci. 2017;32(1):1-28.

3. Moraga P. Geospatial Health Data: Modeling and Visualization with R-INLA and Shiny2019.

4. Cici Chen AYK, Michelle Ross, Jon Wakefield, Mikael Moise SpatialEpi: Methods and Data for Spatial Epidemiology. 1.2.7 ed2021.

5. Blangiardo M, Cameletti M, Baio G, Rue H. Spatial and spatio-temporal models with R-INLA. Spatial and Spatio-temporal Epidemiology. 2013;4:33-49.

## Figure S1. The Netherlands spatial connectivity between a) municipality level, and b) public health services (GGD) level.

## Note: The connectivity between municipalities and public health services regions supports and guides the modelling assumption that areal characteristics are more similar based on proximity rather than distance. For names and more details on the municipalities in the Netherlands please see: <https://www.government.nl/topics/municipalities>; and for Public Health Services regions in the Netherlands, please see: <https://www.ggd.nl/>.

**
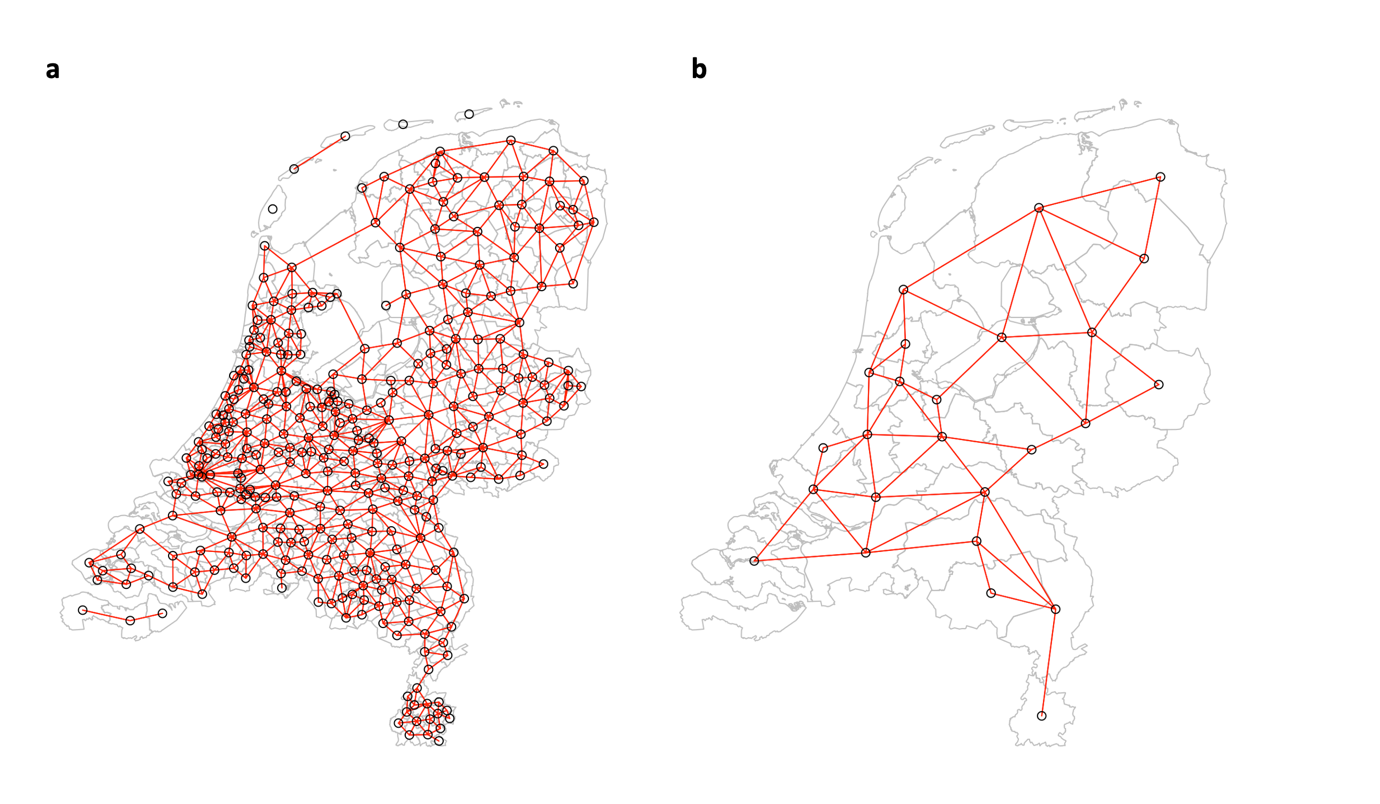
**

## S2 Spatio-temporal distribution of the COVID-19 uptake covered primary partly (only one dose administrated) on the municipality level


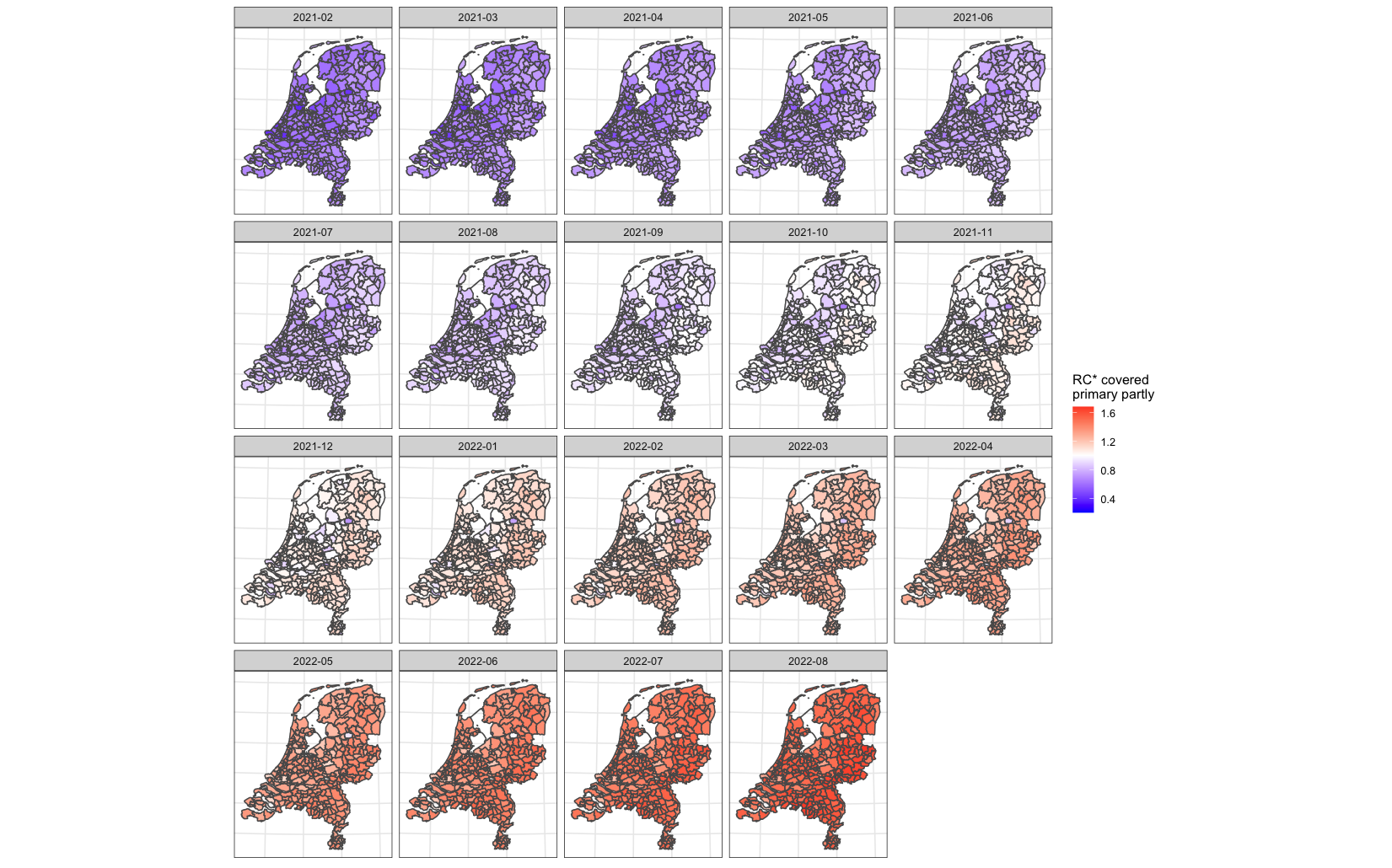


## S3 Spatio-temporal distribution of the COVID-19 uptake covered primary partly (only one dose administrated) on the public health services (GGD) level


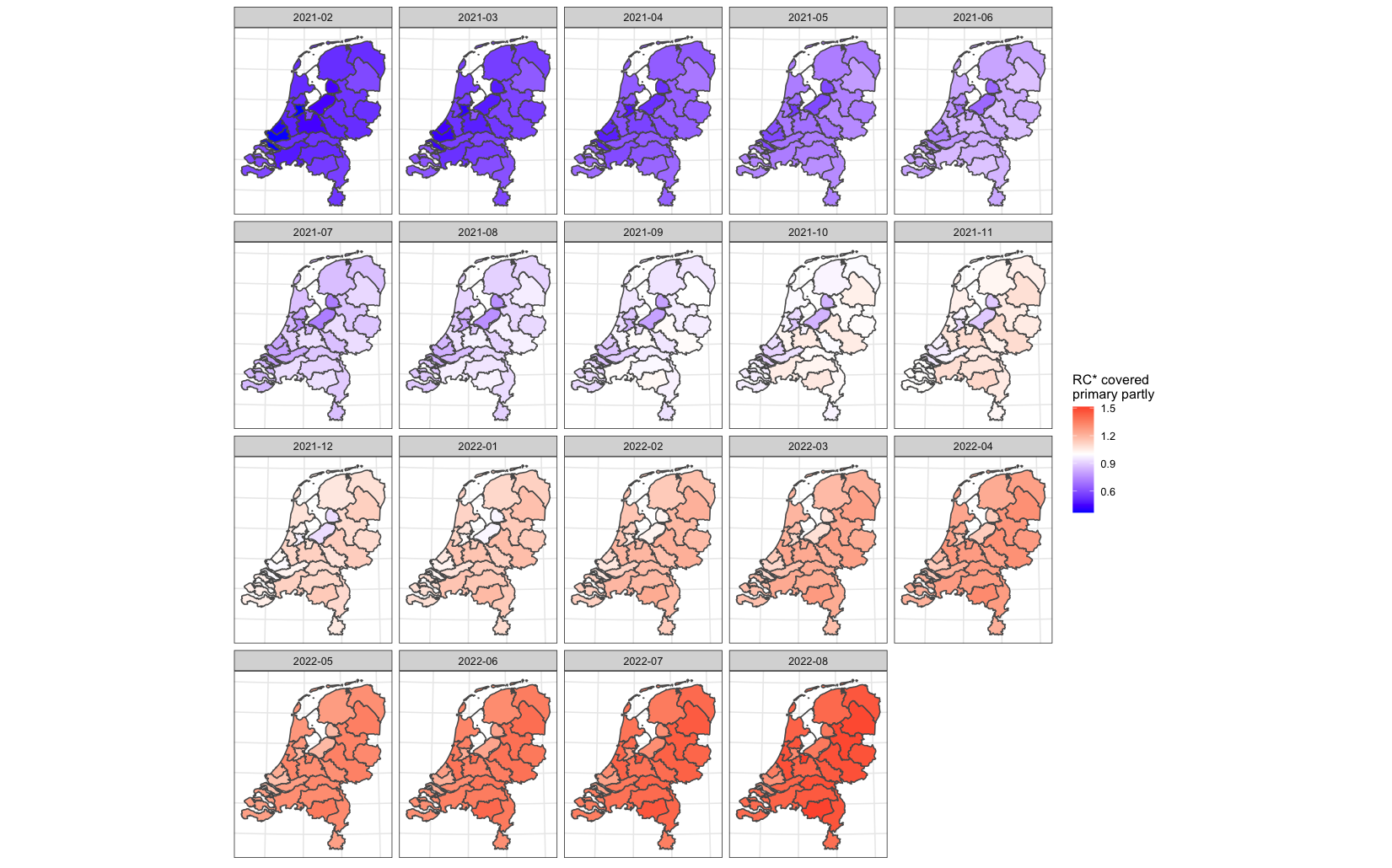


## S4 Spatio-temporal distribution of the COVID-19 uptake covered primary completed (two doses administrated) on the municipality level


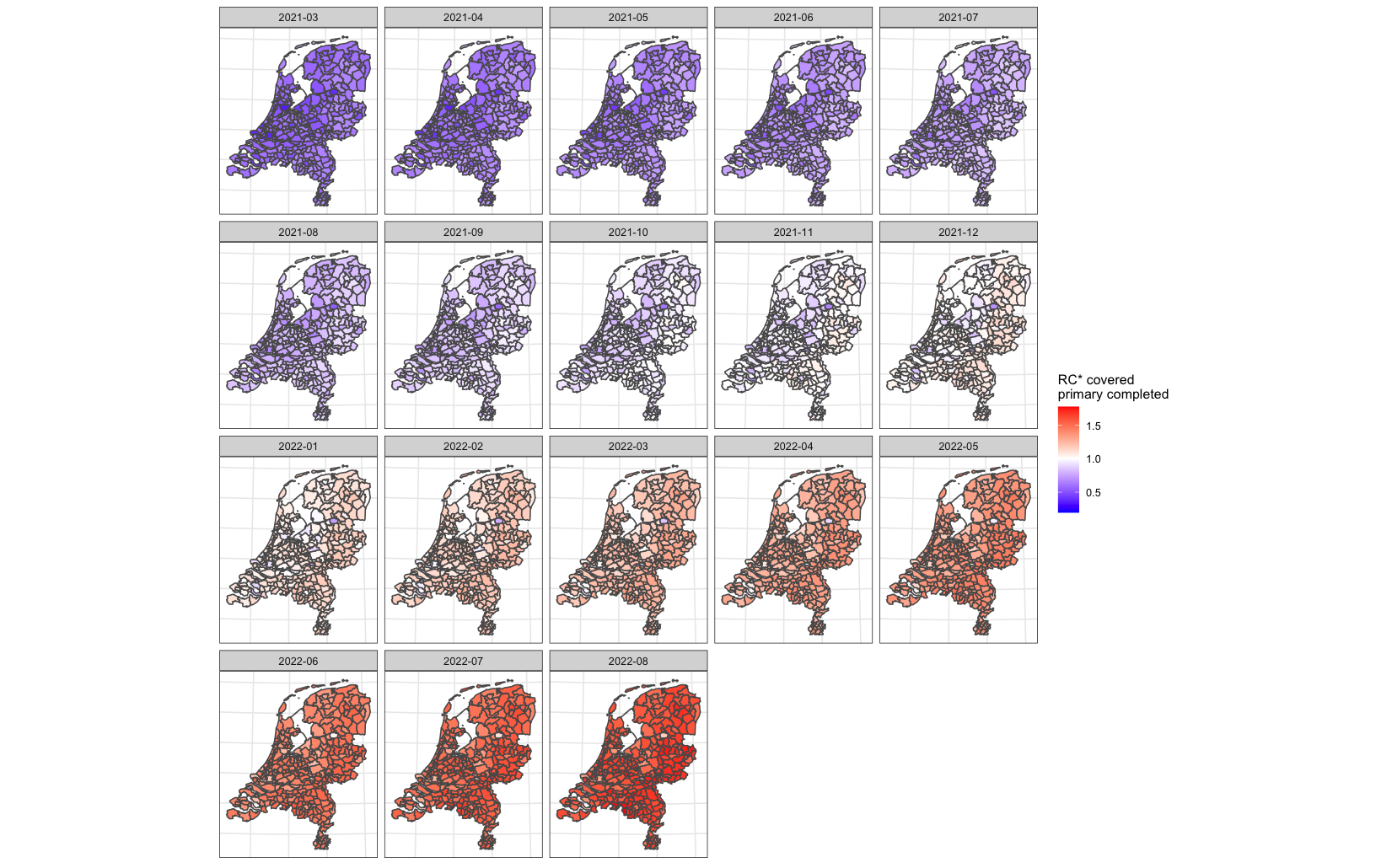


## S5 Spatio-temporal distribution of the COVID-19 uptake covered primary completed (two doses administrated) on the public health services (GGD) level


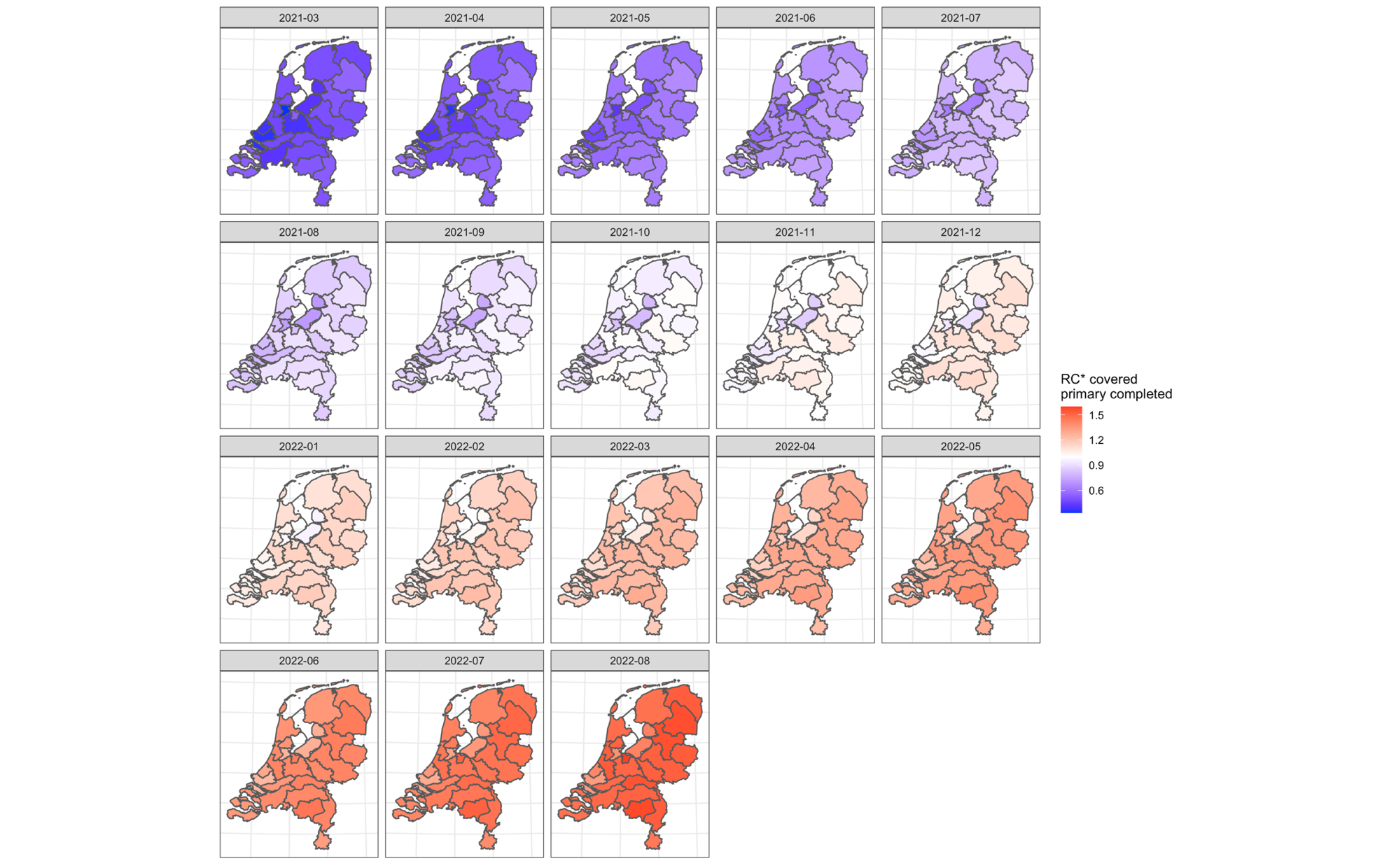


## S6 Spatio-temporal distribution of the COVID-19 uptake covered first booster (one booster dose administrated) on the municipality level


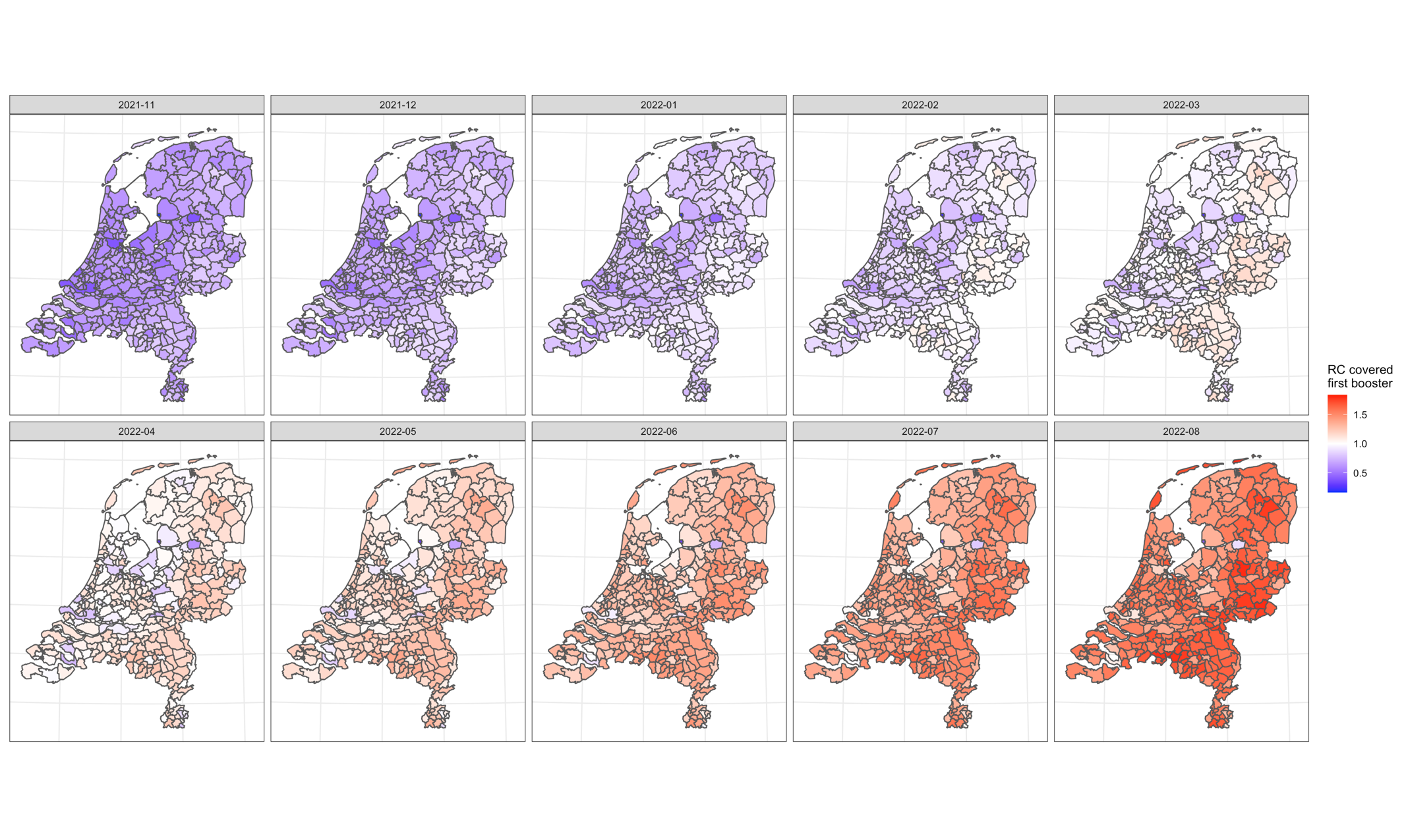


## S7 Spatio-temporal distribution of the COVID-19 uptake covered first booster (one booster dose administrated) on the public health services (GGD) level


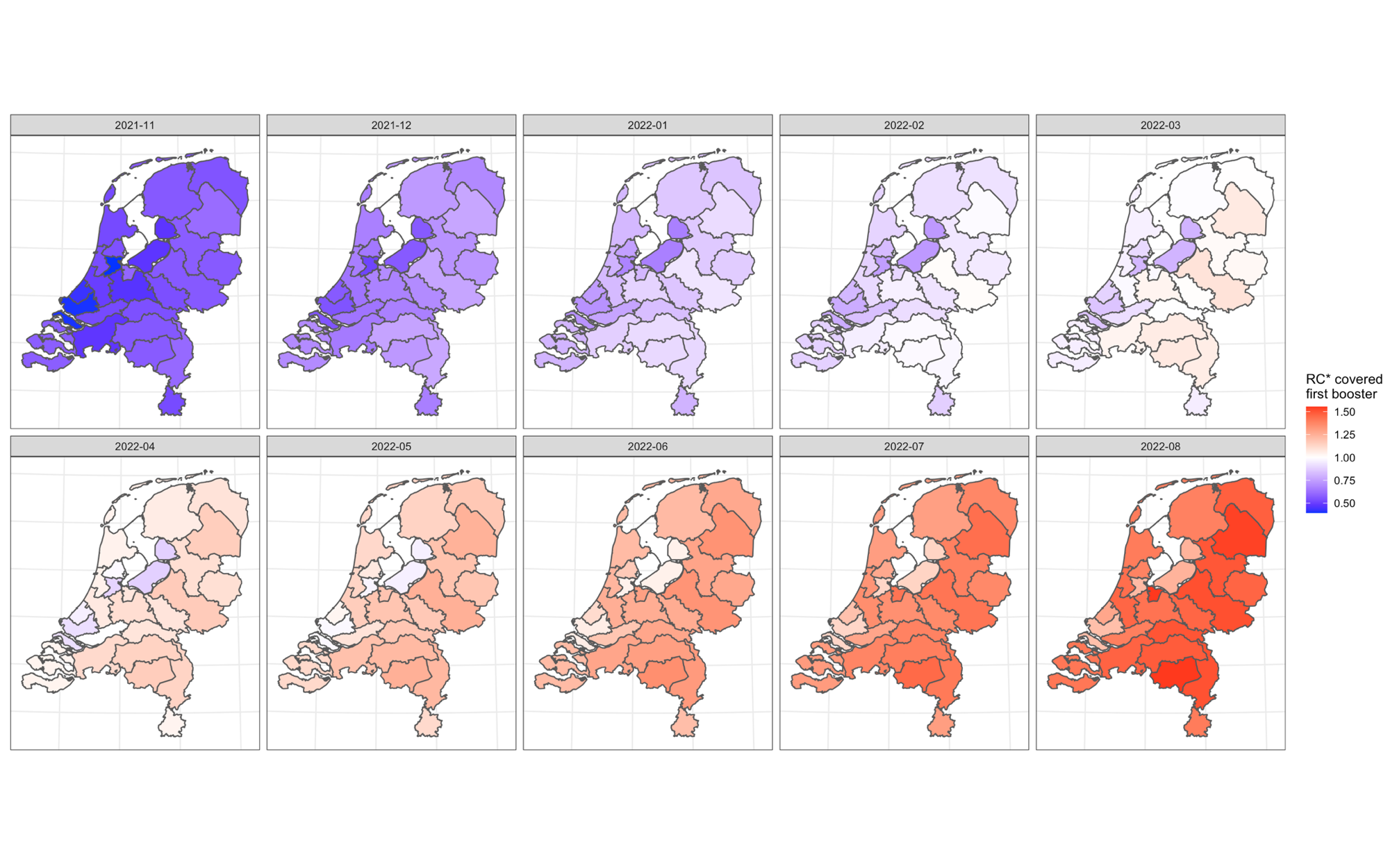


## S8 Spatial distribution of the COVID-19 uptake covered primary partly (only one dose administrated) on the municipality level over the selected periods


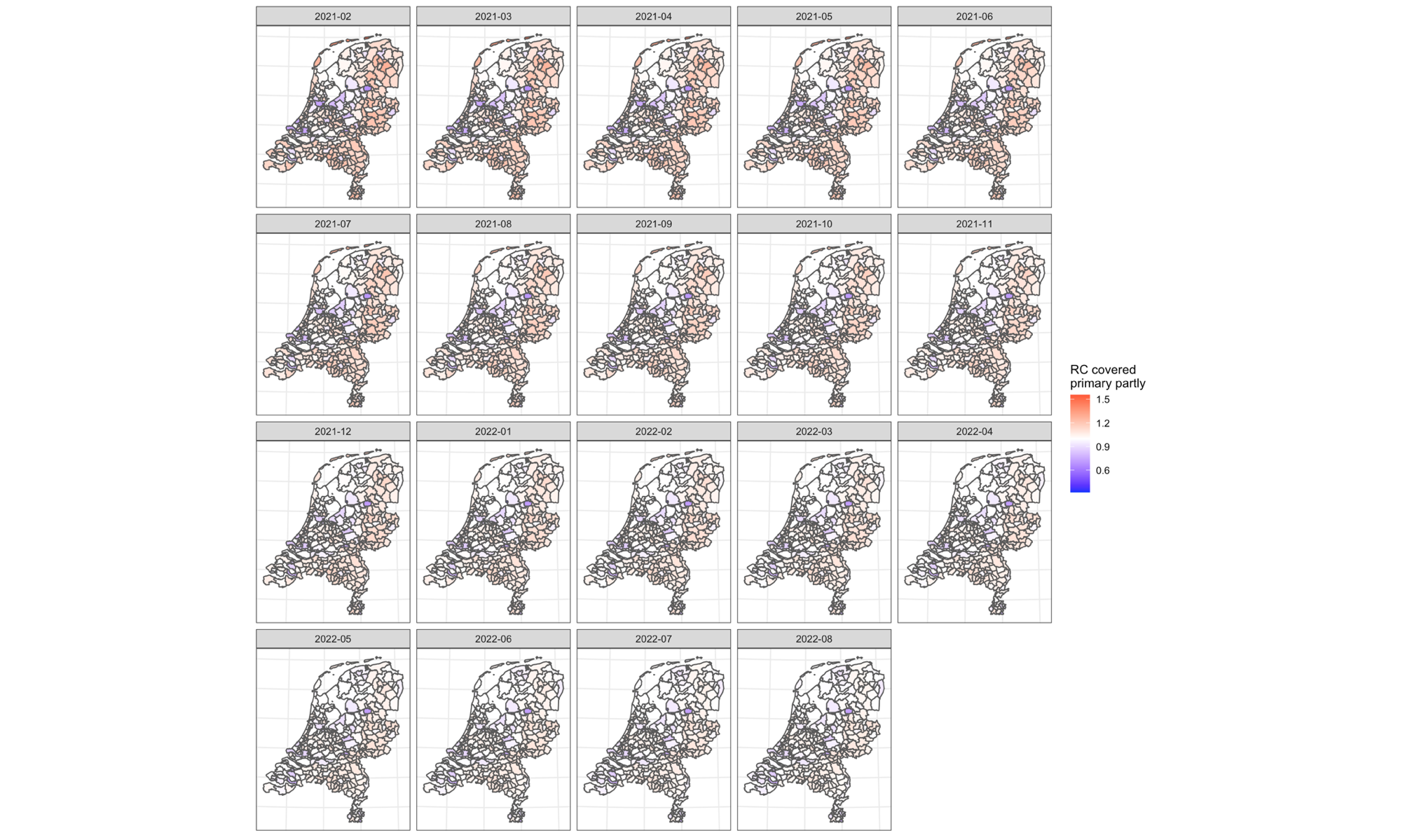


## S9 Spatial distribution of the COVID-19 uptake covered primary partly (only one dose administrated) on the public health services (GGD) level over the selected periods


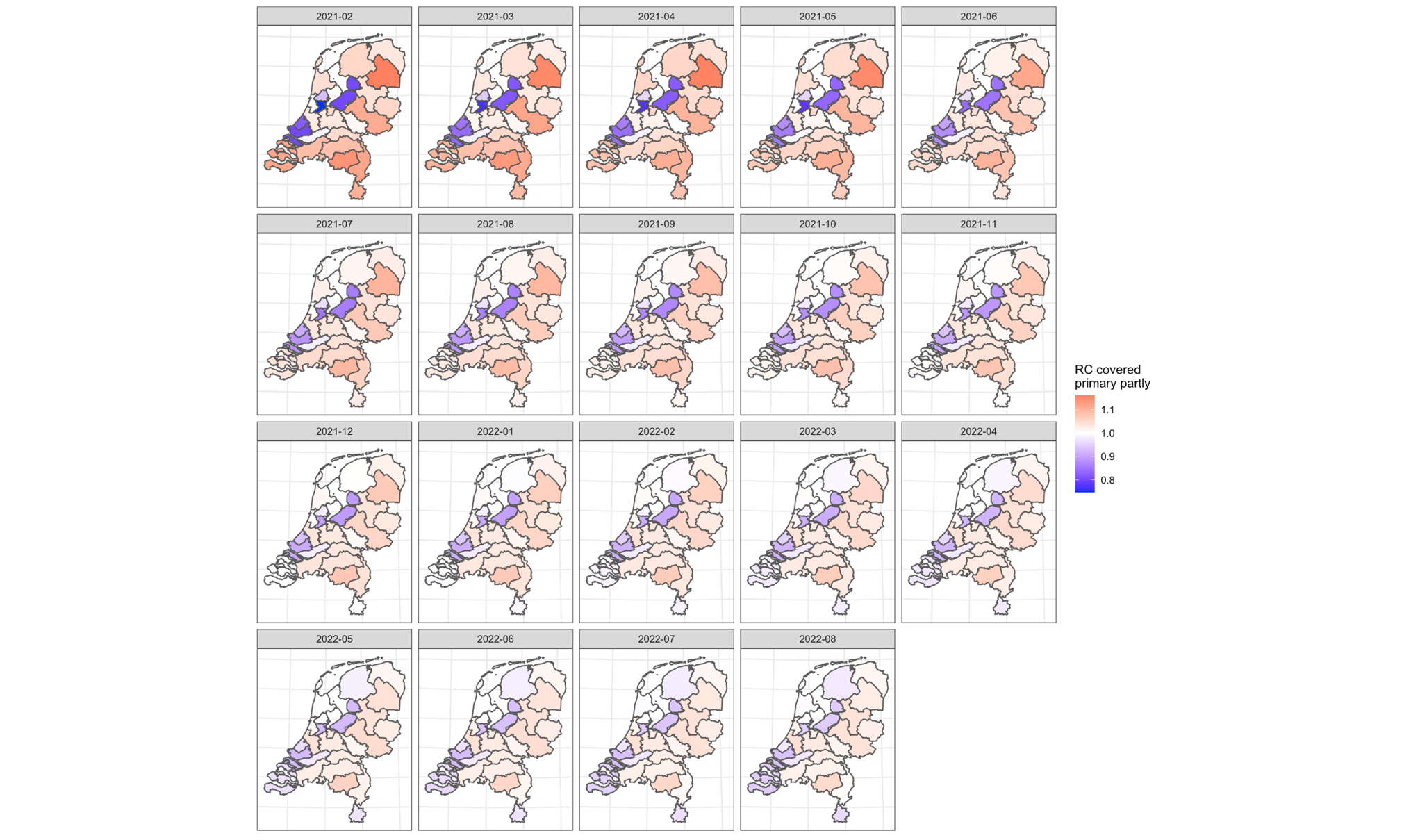


## S10 Spatial distribution of the COVID-19 uptake covered primary completed (two doses administrated) on the municipality level over the selected periods


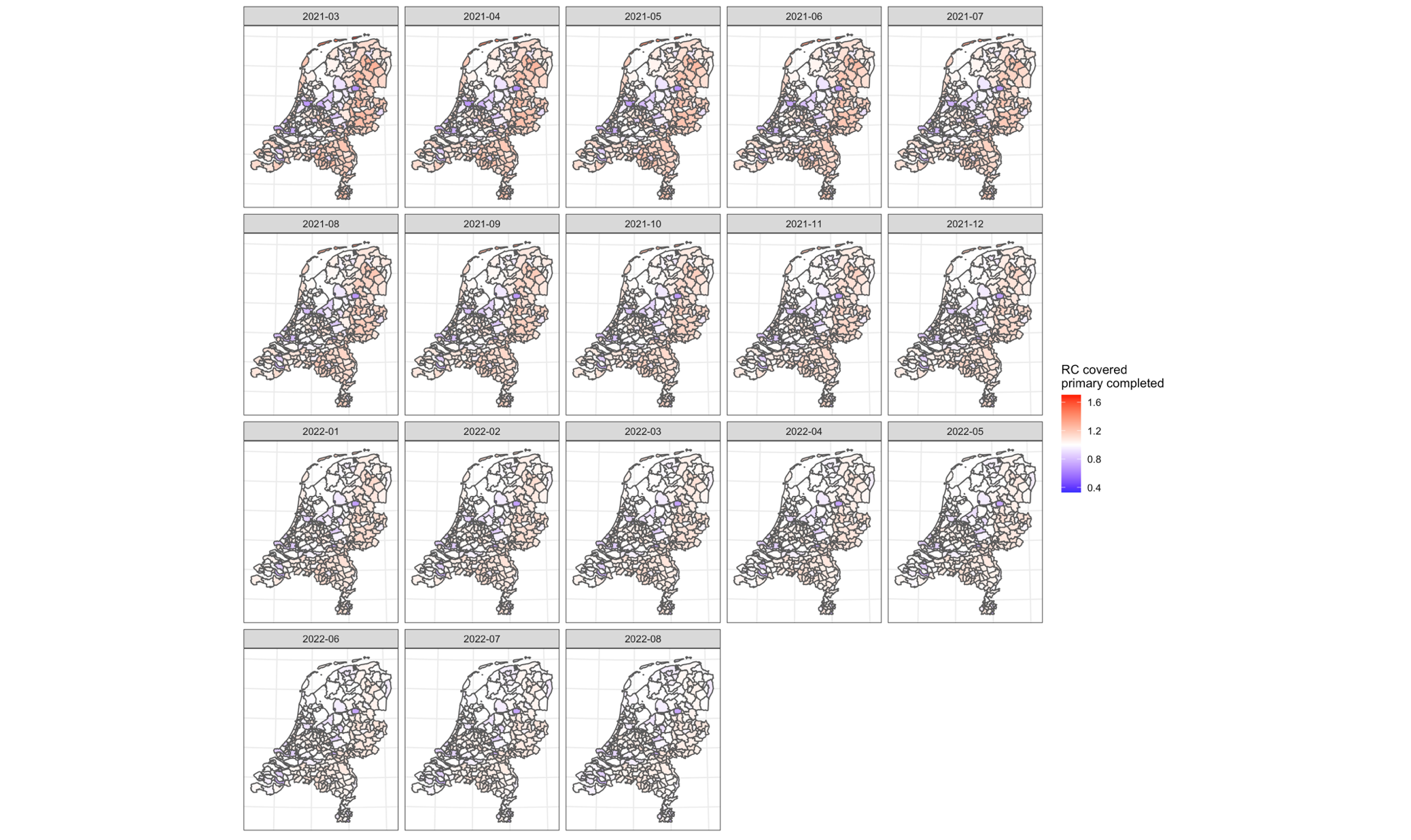


## S11 Spatial distribution of the COVID-19 uptake covered primary completed (two doses administrated) on the public health services (GGD) level over the selected periods


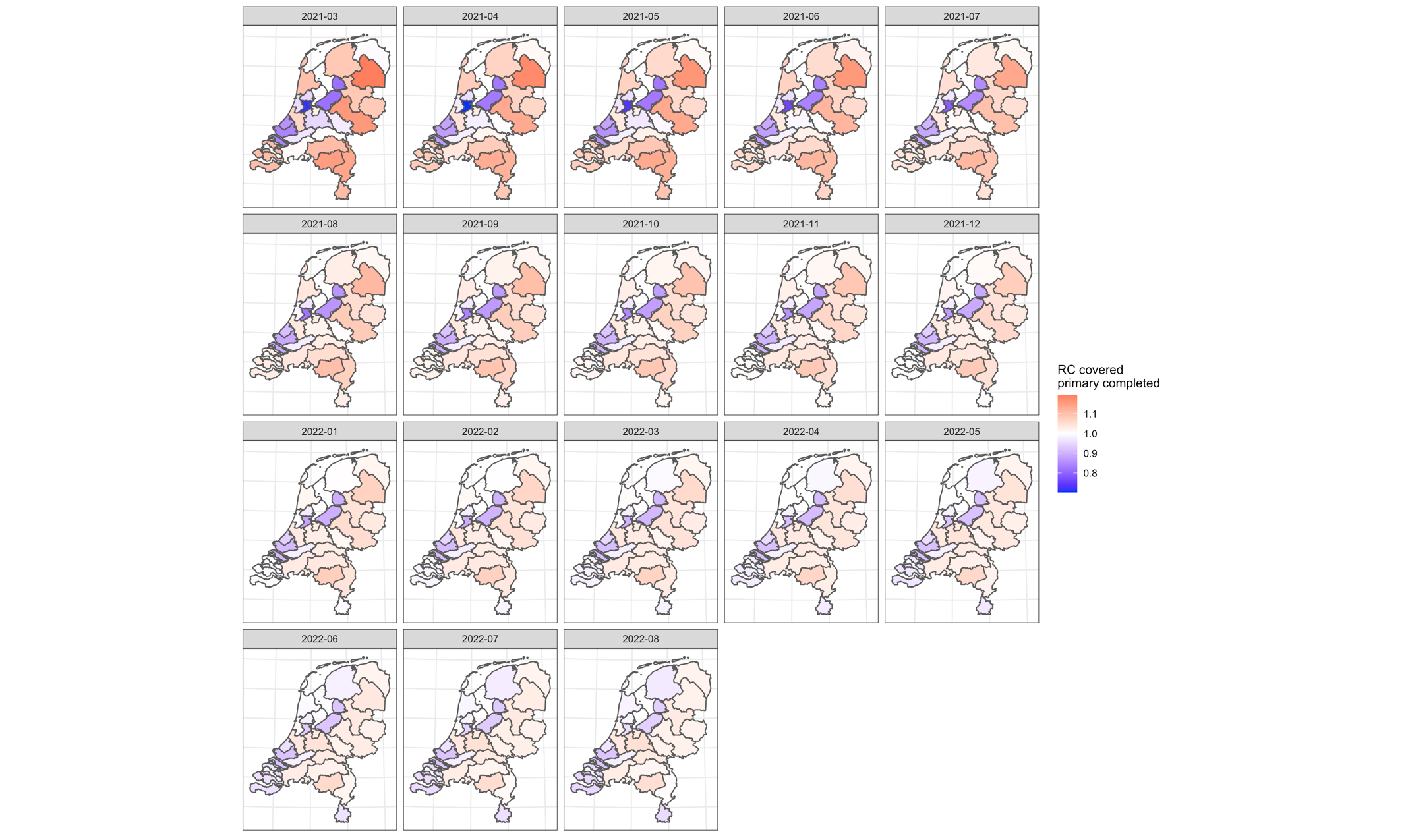


## S12 Spatial distribution of the COVID-19 uptake covered first booster (one booster dose administrated) on the municipality level over the selected periods


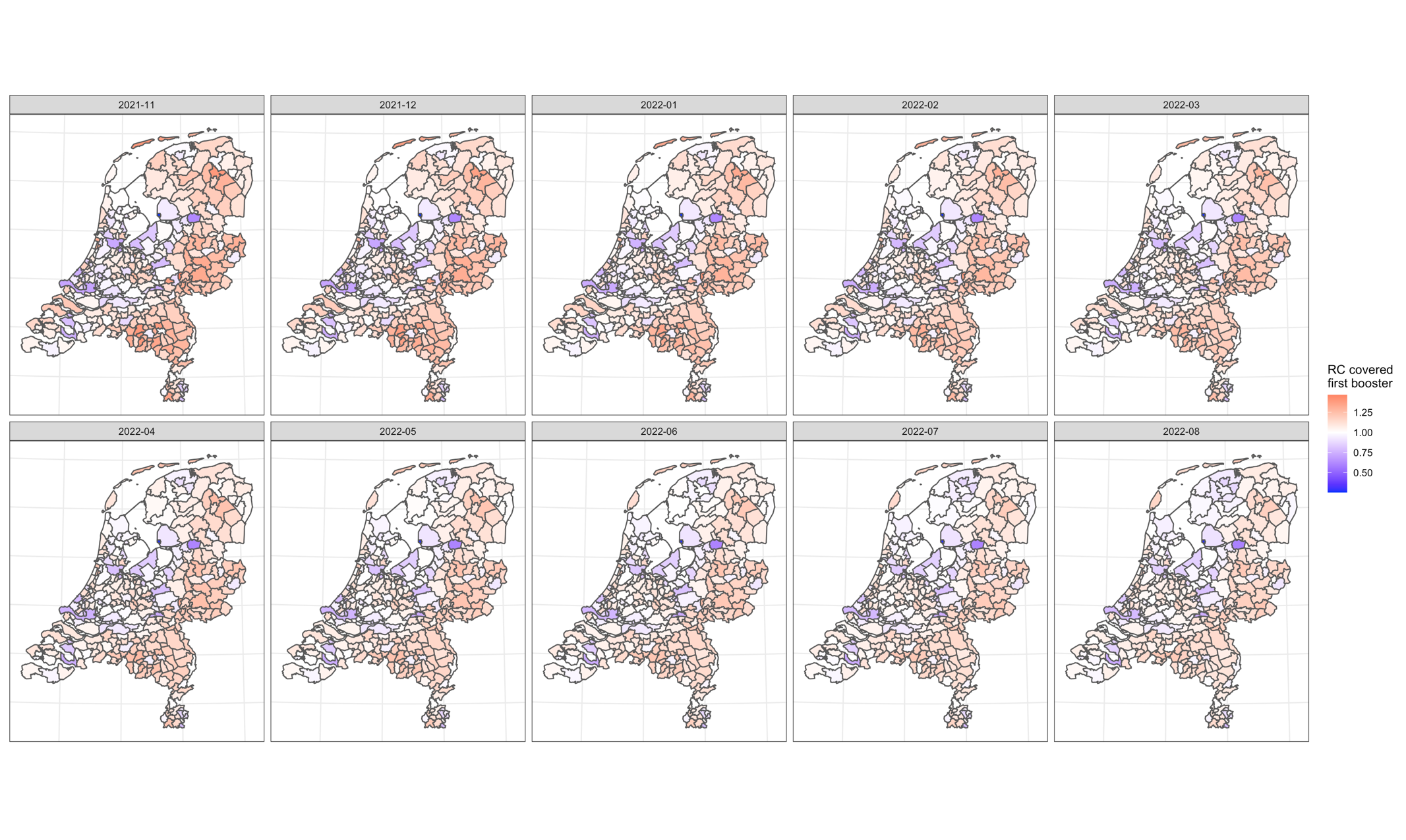


## S13 materials Spatial distribution of the COVID-19 uptake covered first booster (one booster dose administrated) on the public health services (GGD) level over the selected periods


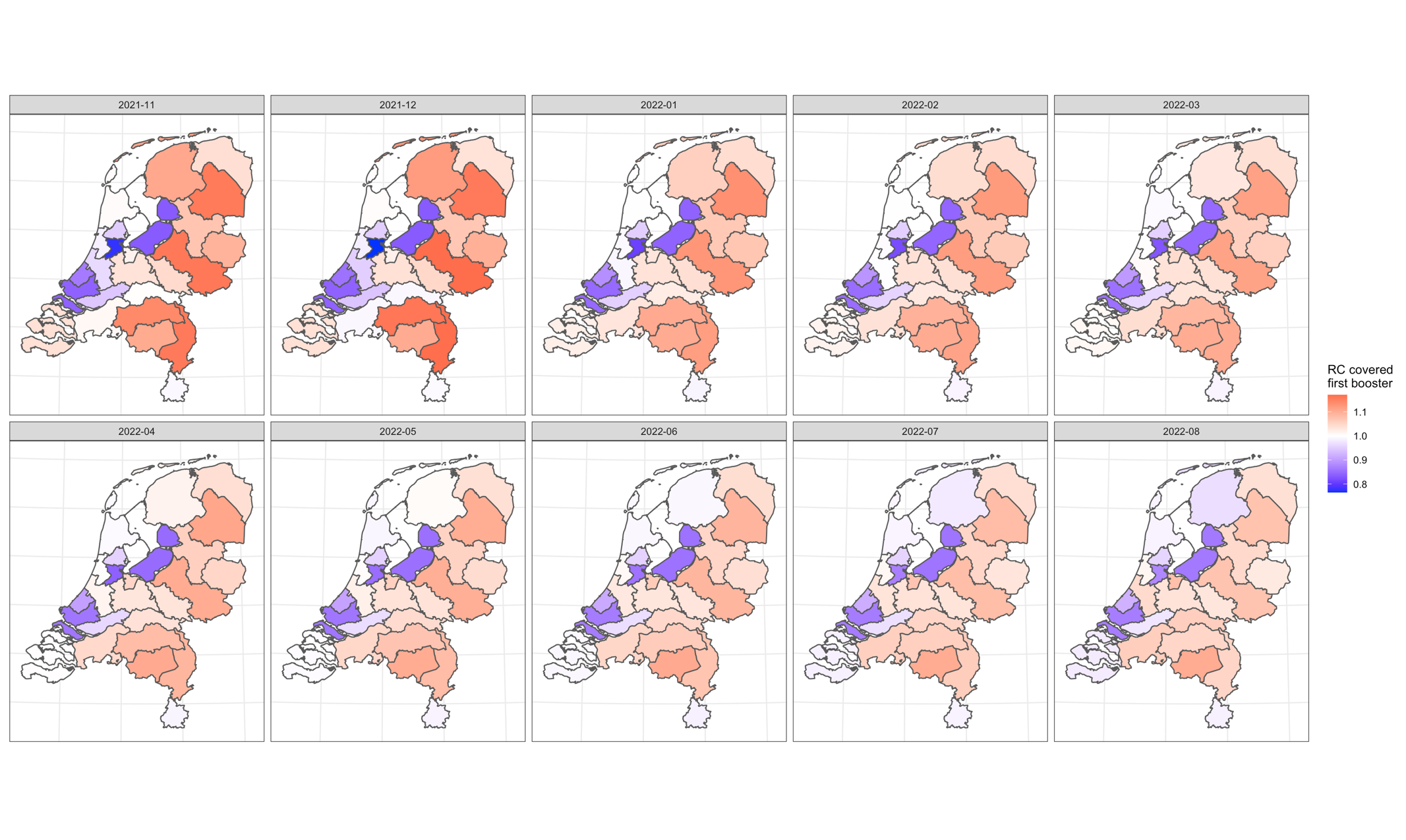

Supplement: Wang et al. supplementary material 3 — Wang et al. supplementary material [file S0950268824001249sup003.docx]
